# Memory B cells and their transcriptomic profiles associated with belimumab resistance in systemic lupus erythematosus in the maintenance phase

**DOI:** 10.3389/fimmu.2025.1506298

**Published:** 2025-02-05

**Authors:** Takeshi Iwasaki, Hajime Yoshifuji, Koji Kitagori, Shuji Sumitomo, Shuji Akizuki, Ran Nakashima, Hideaki Tsuji, Ryosuke Hiwa, Mirei Shirakashi, Kosaku Murakami, Akira Onishi, Hideo Onizawa, Masao Tanaka, Fumihiko Matsuda, Akio Morinobu, Koichiro Ohmura

**Affiliations:** ^1^ Graduate School of Medicine, Kyoto University, Department of Rheumatology and Clinical Immunology, Kyoto, Japan; ^2^ Graduate School of Medicine, Kyoto University, Center for Genomic Medicine, Kyoto, Japan; ^3^ Kobe City Medical Center General Hospital, Department of Rheumatology, Kobe, Japan; ^4^ Division of Clinical Immunology and Cancer Immunotherapy, Center for Cancer Immunotherapy and Immunobiology, Graduate School of Medicine, Kyoto University, Kyoto, Japan; ^5^ Department of Advanced Medicine for Rheumatic Diseases, Kyoto University Graduate School of Medicine, Kyoto, Japan

**Keywords:** systemic lupus erythematosus, belimumab, memory B cell, RNA-Seq, omics, maintenance phase

## Abstract

The factors contributing to the treatment efficacy of belimumab in patients with systemic lupus erythematosus (SLE) in the maintenance phase are unknown. Here, we collected blood samples from patients with SLE (n=44) treated with belimumab before and three and six months after treatment. RNA-Seq of whole blood was performed, and gene expression was quantified. Immune cell type enrichment analysis estimated immune cell subtype proportions and gene expression in each subtype. The Systemic Lupus Erythematosus Disease Activity Index 2000 (SLEDAI-2K) < 4 at six months was set as the primary efficacy criterion. Non-responders exhibited upregulated B cell proliferation signals before treatment, associated with an increased number of memory B cells. A higher proportion of memory B cells before treatment predicted poor response (*p*=5.1×10^-4^). This was also associated with changes in disease activity and glucocorticoid dose at six months compared with baseline. Belimumab did not affect memory B cell proportion during the treatment time course, in contrast to naïve B cells. Higher memory B cell proportion was associated with higher type-I interferon (IFN) scores and lower white blood cell and complement C4 levels. Transcriptomic analysis of memory B cells in non-responders revealed significant upregulation of immunoglobulin genes (Ig). Memory B cells and high Ig expression in them were identified as a treatment-resistant factor of belimumab in SLE patients. Lower C4 and white blood cell counts may serve as clinical markers of higher memory B cells.

## 1 Introduction

Systemic lupus erythematosus (SLE) is a chronic autoimmune disorder that affects a variety of organ systems and markedly impairs health-related quality of life (1). Patients with SLE often have increased blood levels of the B lymphocyte stimulator (BLyS; also termed BAFF) (2). It is a member of the tumor necrosis factor ligand family that induces B cell proliferation and immunoglobulin secretion (3). Belimumab is a recombinant, human immunoglobulin G1 lambda monoclonal antibody that binds soluble BAFF protein and inhibits its biological activity (4, 5). In terms of cell type specificity, it has a different mode of action from anifrolumab, which blocks the type I interferon receptor signaling across multiple immune cell types (6).

This drug is currently recommended to be used to achieve disease remission as well as to decrease the dose of oral glucocorticoid (GC) in the maintenance phase (7). However, as with other biological agents, some patients are known to exhibit poor responses to this drug. The recent application of new treatment options, such as anifrolumab, underscores the necessity of developing predictive models for treatment response to belimumab. To date, lower disease activity (5), higher complement (8), lower ds-DNA antibody (8), established organ damage (9), lower BlyS (10, 11), a higher number of B cells (12), a smaller number of differentially expressed genes induced by belimumab treatment (13) are reported to be associated with poor response to this drug.

However, there are several limitations of the previous studies. The first one is that most of those studies focus on high disease activity status. Since patients with high disease activity were reported to show better responses to belimumab (8), non-responders should be enriched in moderate to low disease activity patients. This indicates that focusing on such patients has the potential power to elucidate factors associated with treatment resistance. The second limitation is that few of the previous studies focus on the genome-wide transcription difference between responders and non-responders. A comprehensive approach, such as comparing genome-wide expression profiles, has the potential power to detect unprecedented findings.

In this study, we assessed the treatment resistance factor of belimumab through clinical and molecular level profiling of moderate to low disease activity patients.

## 2 Methods

### 2.1 Study patients

**Figure 1:**
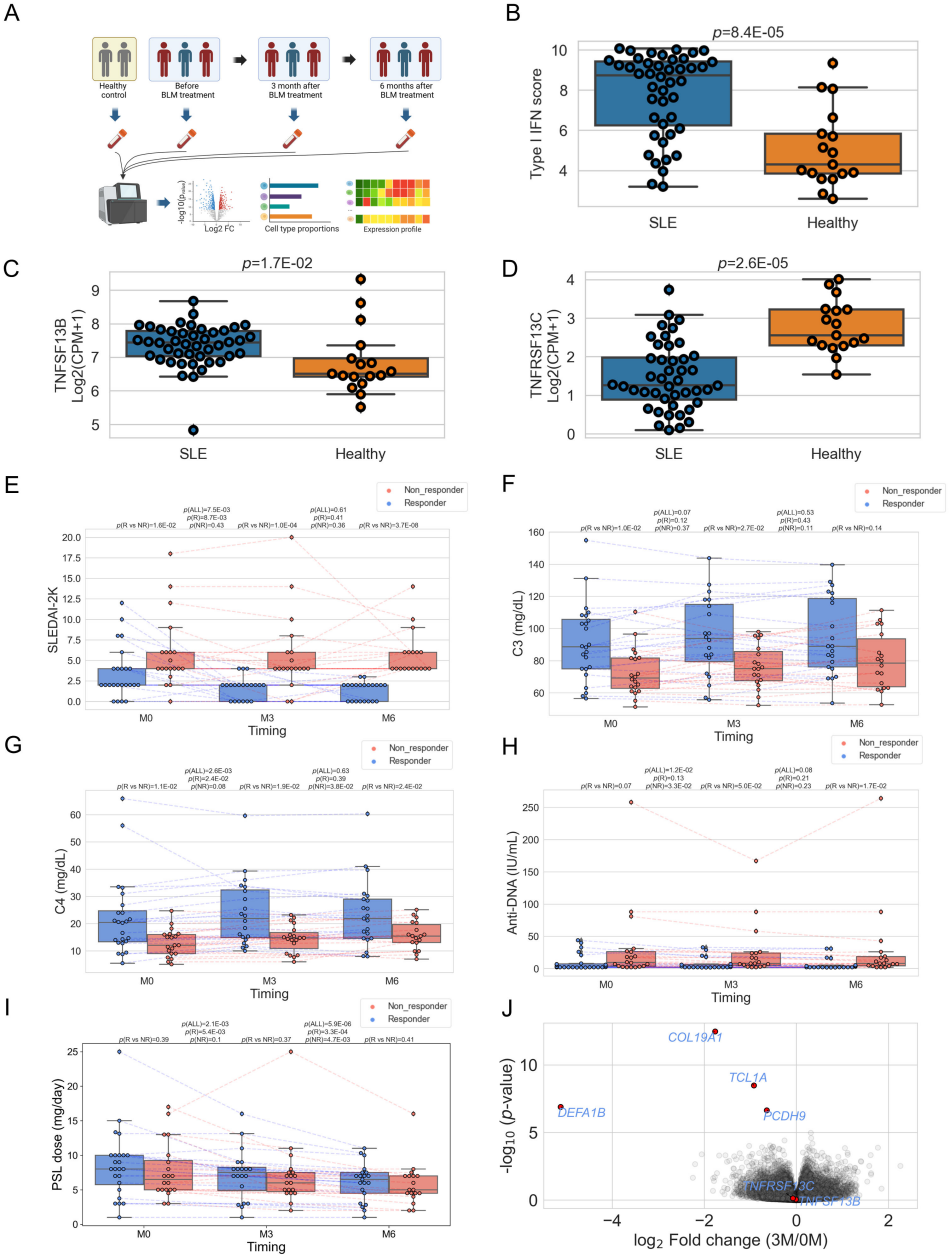
**(A)** Schematic of study design. BLM: belimumab. Created with BioRender.com. Box plots comparing type I IFN score **(B)**, the expression level of *TNFSF13B* (BAFF) **(C)**, and *TNFRSF13C* (BAFF-R) **(D)** between SLE patients and healthy controls. CPM, Count per million **(E–I)** Box plots comparing each clinical index between responders and non-responders and its time course. "M0" stands for before treatment, "M3" for three, and "M6" for six months after treatment. "P(R vs. NR)" represents *P*-value comparing responders and non-responders, "P(ALL)" for the *P*-value comparing paired samples between 0M vs. 3M, or 3M vs. 6M. "P(R)" for *P*-value comparing 0M vs. 3M, 3M vs. 6M within responders, and "P(NR)" stands for the P-value obtained by comparison within non-responders. **(J)** Volcano plots showing differentially expressed genes between before treatment and three months after treatment. In **(E–I)**, each dot represents each specimen.

The study design is shown in **Figure 1A**. We enrolled 46 patients diagnosed with SLE according to the European League Against Rheumatism/American College of Rheumatology classification criteria (The 2019 EULAR/ACR classification criteria) (14). Among the 46 patients, 38 were from Kyoto University Hospital, Kyoto, Japan, and the remaining eight were from Kobe City Medical Center General Hospital, Kobe, Japan. We excluded samples derived from two patients in which belimumab treatment was discontinued before six months due to a change of hospital (n=1) and withdrawal of treatment due to the patient’s request (n=1). We also enrolled healthy individuals (n=17) matched for age and sex (**Supplementary Table 1**).

### 2.2 Clinical evaluation and definition

At every clinic visit, disease activity was evaluated using the systemic lupus erythematosus disease activity index (SLEDAI-2K) (15). Clinical characteristics, including age, sex, treatment profiles (the use of prednisolone (PSL) and immunosuppressive agents), and clinical measurements (white blood cells, hemoglobin, complement) were obtained from medical records. The information on SLICC/ACR damage index (SDI) (15) and information on disease onset was obtained from Kyoto Lupus Cohort (16–18) for 38 samples in Kyoto University Hospital and from Kobe Lupus Cohort for 8 samples in Kobe City Medical Center General Hospital. Patients with a SLEDAI-2K score < 4 after six months of treatment with belimumab were defined as responders; those with a score of 4 or higher were classified as non-responders.

### 2.3 Sample collection, RNA Sequencing, transcriptome analysis

We collected blood samples from patients before and approximately 3 and 6 months after treatment, respectively. There were no missing samples from the before-treatment specimens for 44 patients; however, five samples were missing at the three-month time, and four were missing at the six-month time after treatment. Whole blood samples were stored in PAXgene tubes (QUIAGEN). RNA was extracted by PAXgene Blood RNA Kit (QUIAGEN). Library preparation was performed using TruSeq stranded Total RNA Library PrepKit with Ribo-Zero Globin Human. Sequencing was conducted by NovaSeq6000 in the 100-bp paired-end mode. Sequencing reads were trimmed using Trimmomatic ver. 0.36 (19) (leading: 20, trailing: 20, slidingwindow: 4:15, minlen: 36) and aligned to hg38 reference genome using STAR (ver. 2.7.3a) (20). Gene counts were generated by RSEM (ver. 1.3.1) (21) using Homo_sapiens.GRCh38.95.gtf from the Ensembl database (22). Gene counts were normalized by size factor implemented in DESeq2 (23) and converted to count per million (CPM), and log_2_(CPM+1) was calculated. For the genome-wide differential gene expression test, we filtered low-count genes (0 count in ≥ 30% of 140 specimens, which include specimens from patients and healthy controls) and performed the Wald test using DESeq2. Type I IFN score was calculated by the mean expression of the four genes *(IFI27, IFI44, IFI44L*, and *RSAD2*) (6). Gene set enrichment analysis (24) was performed for 7,658 GO pathways in MSigDB (ver 7.5) (25) with 10,000 permutations. Overrepresentation analysis of gene sets was performed using Metascape (26) with default settings.

### 2.4 Immune cell type enrichment analysis

We performed an immune cell type enrichment analysis using CIBERSORTx (27) to estimate the proportion of each of the 22 cell types and the gene expression in each cell type in each specimen, utilizing the leukocyte signature matrix (LM22) (28) as a reference panel. Differential cellular proportion tests between responders and non-responders were performed on 13 cell subtypes (**Table 1**), excluding nine cell types (Mast cells activated, Dendritic cells resting, T cells follicular helper, T cells regulatory (Tregs), Eosinophils, Macrophages M0, Macrophages M1, Macrophages M2, and T cells gamma delta) whose estimated proportion was zero in over 95% of specimens (>133/140 specimens). We imputed expression of 57,189 genes (all genes quantified by RSEM) in memory B cells for each specimen using high resolution (‘hires’) function of CIBERSORTx, utilizing the same reference panel (LM22). Differential gene expression tests between responders and non-responders in memory B cells were performed on 4,154 genes, excluding 53,035 genes whose expression was estimated to be the same value or “NA” in all specimens. The Mann-Whitney test was used, and false discovery rate (FDR) was calculated by the Benjamini-Hochberg method.

**Table 1:** Comparison between responders and non-responders.

Name	Before treatment			Three months after treatment			Six months after treatment		
	Responder	Non-responder	P value	Responder	Non-responder	P value	Responder	Non-resonder	P value
Age (years)	45.467 (38.62-59.8)	37.125 (29.42-48.93)	0.08						
Disease duration (years)	17.008 (11.54-29.1)	9.295 (6.31-17.03)	0.06						
Female (%)	21 (87.5)	19 (95.0)	0.61						
Use of immunosuppressive agents (%)	19 (82.6)	19 (95)	0.35						
SDI	3.0 (2.0-5.0)	2.0 (1.0-2.25)	0.03						
SLEDAI-2K	2.0 (2.0-4.0)	4.0 (4.0-6.0)	0.02	2.0 (0.0-2.0)	4.0 (4.0-6.0)	1.0E-04	2.0 (0.0-2.0)	4.0 (4.0-6.0)	3.7E-08
Prednisolone dose, median (mg/day)	8.0 (5.75-10.0)	6.5 (5.0-9.25)	0.39	7.5 (4.88-8.25)	6.0 (4.75-7.5)	0.37	6.75 (4.62-7.38)	5.0 (4.5-7.0)	0.41
White blood cell (×10^3^/μL)	7.31 (4.98-10.29)	5.175 (4.14-6.67)	0.07	6.11 (4.79-8.7)	5.7 (4.62-7.67)	0.72	7.92 (4.52-9.04)	5.105 (4.48-5.42)	0.40
Hb (g/dL)	12.2 (10.95-13.45)	12.9 (10.77-13.52)	0.71	12.2 (10.7-13.4)	12.3 (10.7-13.6)	0.94	11.95 (10.95-13.45)	11.75 (10.7-12.95)	0.81
Platelet (×10^3^/μL)	245.0 (186.5-281.5)	215.0 (188.75-242.25)	0.38	240.0 (174.0-287.0)	217.0 (179.5-233.5)	0.56	254.5 (179.0-285.25)	249.0 (202.0-281.25)	0.79
C3 (mg/dL)	88.65 (74.85-104.5)	69.2 (62.8-81.65)	0.01	94.6 (81.8-114.0)	75.9 (71.15-81.5)	0.03	89.0 (76.9-121.18)	74.75 (65.35-98.0)	0.14
C4 (mg/dL)	20.45 (13.25-26.1)	12.1 (9.0-16.05)	0.01	22.9 (15.1-33.5)	14.4 (11.5-18.1)	0.02	21.55 (16.58-30.5)	15.45 (13.4-16.0)	0.02
CH50/mL	44.0 (31.1-46.0)	32.0 (24.95-35.33)	0.01	44.0 (39.0-51.0)	31.6 (29.45-35.5)	2.6E-03	45.0 (34.55-54.0)	33.85 (31.4-42.0)	0.09
IgG (mg/dL)	945.0 (869.0-1074.0)	1288.5 (942.0-1547.5)	0.03	965.0 (928.0-1227.0)	1407.0 (1010.0-1597.5)	0.30	918.5 (710.0-1036.75)	1236.5 (1160.25-1346.5)	0.058
IFN score	5.332 (4.93-5.55)	5.444 (5.26-5.59)	0.22	5.365 (4.99-5.5)	5.416 (5.23-5.7)	0.32	5.306 (5.04-5.52)	5.404 (5.09-5.71)	0.27
Anti-DNA (IU/mL, RIA)	3.0 (2.0-21.0)	17.5 (5.9-28.0)	0.07	3.4 (2.0-18.0)	16.0 (7.5-32.5)	0.05	2.0 (1.85-4.15)	11.0 (5.0-26.0)	0.02
B cells naïve (%)	0 (0-7.6E-03)	0 (0-0.21)	0.55	0 (0-0)	0 (0-3.5E-03)	0.39	0 (0-0)	0 (0-0)	0.19
B cells memory (%)	0 (0-0.47)	0.95 (0.69-1.63)	5.1E-04	0.33 (4.4E-02-0.91)	0.62 (0-1.57)	0.72	0.13 (0-0.79)	0.64 (0.11-1.03)	0.20
Plasma cells (%)	0.35 (0.14-0.74)	0.21 (0.09-0.85)	0.54	0.21 (0.1-0.66)	0.23 (0-0.77)	0.88	0.26 (0.16-0.85)	0.37 (0.18-0.66)	1.00
T cells CD8 (%)	0.63 (0-1.92)	1.74 (1.2-2.96)	0.13	1.42 (0.59-3.15)	1.65 (0.76-3.58)	0.57	1.32 (0-3.37)	3.37 (0.9-5.14)	0.13
T cells CD4 naïve (%)	9.68 (6.2-11.5)	12.15 (8.55-14.65)	0.04	9.49 (7.3-14.25)	11.72 (9.66-15.81)	0.19	10.86 (7.69-15.77)	14.56 (8.51-18.59)	0.23
T cells CD4 memory resting (%)	7.1 (3.3-10.2)	8.15 (4.89-9.36)	0.47	7.06 (5.47-8.81)	6.89 (5.09-10.18)	0.56	6.92 (4.83-9.52)	8.05 (5.68-9.23)	0.56
T cells CD4 memory activated (%)	1.41 (0.67-2.73)	0.9 (0.31-1.48)	0.08	1.29 (0.68-1.83)	1.01 (0.36-1.41)	0.28	1.35 (0.53-2.34)	0.82 (0.34-1.03)	0.08
NK cells resting (%)	5.26 (3.57-6.56)	4.77 (3.07-5.54)	0.26	5.67 (3.22-7.83)	3.86 (2.36-5.33)	0.06	5.6 (3.66-7.63)	4.04 (3.1-6.58)	0.30
NK cells activated (%)	0 (0-0)	0 (0-0)	0.91	0 (0-0)	0 (0-0)	0.98	0 (0-0)	0 (0-0)	0.19
Monocytes (%)	25.73 (19.0-30.11)	22.32 (17.18-26.91)	0.35	24.89 (18.36-31.97)	24.55 (17.71-31.66)	0.75	27.37 (18.58-32.46)	22.27 (14.39-24.36)	0.08
Dendritic cells activated (%)	0.45 (0.29-0.66)	0.44 (0.34-0.56)	0.90	0.46 (0.24-0.6)	0.48 (0.36-0.73)	0.39	0.48 (0.37-0.68)	0.42 (0.17-0.49)	0.14
Mast cells resting (%)	1.69 (0.88-2.23)	1.88 (1.27-2.17)	0.63	1.44 (0.85-1.85)	1.82 (0.93-2.35)	0.18	1.66 (0.68-2.22)	1.26 (0.54-1.85)	0.47
Neutrophils (%)	44.71 (40.47-57.02)	51.82 (39.83-57.31)	0.97	42.25 (34.87-56.99)	41.33 (32.47-54.23)	0.62	43.68 (29.37-51.96)	44.67 (35.17-56.62)	0.78

Median (IQR) values are shown for continuous variables. The Mann-Whitney U test was used to test the significance.

### 2.5 Single-cell data analysis

To compare the expression levels of Ig within memory B cells across cell subpopulations, publicly available single-cell data of human peripheral blood mononuclear cells (PBMC) (29) were downloaded, comprising both CITE-Seq and scRNA-Seq data. Specifically, data from cells annotated as memory B cells (n=3,285) were utilized. Each immune subset was defined as follows: unswitched memory B cell (CD27^+^IgD^+^), switched memory B cell (CD27^+^IgD^-^), non-activated memory B cell (CD27^+^CD38^lo^), and activated memory B cell (CD27^+^CD38^hi^). The definitions of CD27^+/-^ and IgD^+/-^ were based on whether cellular protein levels were nonzero or zero. Regarding CD38, data from two antibodies (“CD38-1” and “CD38-2”) were available, and we analyzed “CD38-1” data, as the data of CD38-1 and CD38-2 were well-correlated within memory B cells (data not shown), and no superiority was observed between them. The cutoff line for CD38-high (CD38^hi^) or CD38-low (CD38^lo^) cells was determined based on the median value (15) of protein levels in memory B cells. Ig expression levels were calculated as the average expression levels of *IGHM, IGHA2, IGHG2, IGHA1*, and *IGHG1.*

### 2.6 Statistical test

Unless otherwise specified, significant differences were tested using the Wilcoxon signed rank test for the two groups with paired samples and the Mann-Whitney test for the two groups with unpaired samples. The analysis of the usefulness of identified variables in distinguishing responders from non-responders employed receiver-operating-characteristic (ROC) curve techniques. Basically, we calculated the true/false positive rate and cutoff value to distinguish non-responders from responders; however, as for white blood cell count and C4, because the values were lower in non-responders, we calculated cutoff values to detect responders from non-responders. An area under the curve (AUC) below 0.6 was considered worthless, between 0.6 and 0.7 as poor, between 0.7 and 0.8 as fair, between 0.8 and 0.9 as good, and above 0.9 as excellent predictive accuracy. The statistical analysis and test were performed by the Python package sklearn (ver 0.23.2) or scipy (ver. 1.5.2).

## 3 Results

### 3.1 Patient characteristics and the effect of belimumab on the clinical and transcriptome feature

A total of 44 belimumab-naïve SLE patients were analyzed in this study (see Methods). Approximately 90% were female, with a median disease duration of 13.5 years (interquartile range (IQR) 6.0-23.5) (**Supplementary Table 1**). All patients were receiving GC treatment (PSL dose 7.5 (5.0-10.0) mg/day), with SLEDAI-2K indicating low to moderate disease activity (median 4.0, IQR 2.0-6.0). Additionally, to examine the transcriptomic features before belimumab administration, we compared the type I IFN score and the expression of *TNFSF13B* (BAFF) and *TNFRSF13C* (BAFF-R) with healthy individuals. The results showed higher type I IFN score and higher expression and *TNFSF13B* and lower expression of *TNFRSF13C* compared to healthy controls, consistent with previous reports (30, 31) and reflecting the characteristics of SLE (**Figures 1B–D**). Subsequently, we observed changes in clinical traits after belimumab administration in these patient groups (**Figures 1E–I** and **Supplementary Figure 1**). Initially, at three months post-administration, SLEDAI-2K decreased, while CH50 and C4 increased, and anti-DNA decreased within all the patients (**Figures 1E, G, H**; **Supplementary Figure 1**). However, from three to six months post-administration, there were no significant changes in these parameters. At six months, whether SLEDAI-2K < 4 or not divided the patient group into approximately halves (SLEDAI-2K < 4: 24/44, 54.5%) (**Figure 1E**). Therefore, we used SLEDAI-2K<4 at six months post-belimumab administration as the primary efficacy criterion (Responders: n=24, Non-responders: n=20). Non-responders had higher baseline SLEDAI-2K and lower SDI, and higher IgG and lower C3, C4, and CH50 levels among laboratory findings compared to responders (**Table 1**). We also tested post-administration changes unique to responders or non-responders. The responders showed a decrease of SLEDAI-2K and an increase of C4 at three months (*p*=0.009, 0.003, respectively, **Figures 1E, G**), while non-responders showed a reduction of anti-DNA antibody titers at three months (*p*=0.033, **Figure 1H**). However, the PSL dosages were reduced in all groups at all time points except for the non-responders at three months. We then observed changes in the transcriptome because of belimumab administration utilizing both responders and non-responders data, finding significant reductions in the expression levels of four genes (*COL19A1, DEFA1B, TCL1A*, and *PCDH9*) three months post-treatment compared to pre-treatment (FDR<0.05) (**Figure 1J**; **Supplementary Table 2**). We investigated the expression of the four genes among immune cell subtypes using the Database of Immune Expression (32) (**Supplementary Figure 2**). We found that *COL19A1, TCL1A*, and *PCDH9* were genes with high expression levels in B cells. On the other hand, *DEFA1B* is poorly expressed in immune cells in physiological states, but it has been reported to be highly expressed in SLE patients (33). Similary, we observed changes between three months and six months utilizing both responders and non-responders, but there were no significant gene expression changes (**Supplementary Table 3**).

### 3.2 B cell proliferation pathway was upregulated in non-responders before treatment

**Figure 2:**
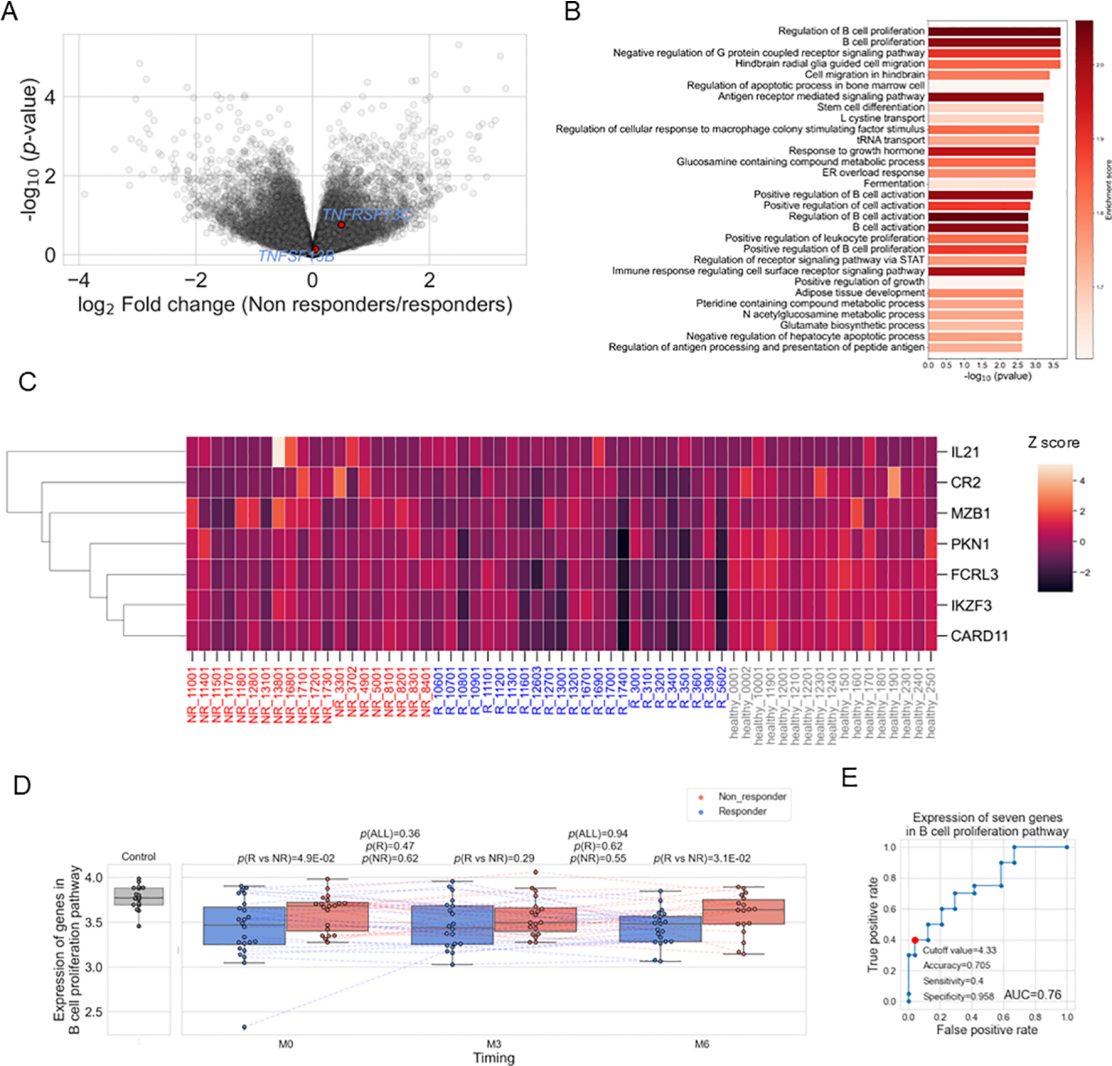
**(A)** Volcano plots showing different expressed genes between responders (R) and non-responders (NR) before treatment. **(B)** Results of gene set enrichment analysis before treatment. **(C)** Heatmap showing the expression of the seven genes in each sample. Expression was standardized across all samples, and the Z scores were shown. **(D)** Box plots comparing the average expression of the 100 genes included in the gene set "GOBP_B_CELL_PROLIFERATION" and their time course. Each dot represents each specimen. Red and blue boxes represent NR and R groups. As for the P values, please refer to the legend of **Figure 1**. **(E)** ROC curve for non-response to belimumab. Red plots indicate the cut-off point at the highest accuracy for predicting non-responders.

Next, we conducted differential expression gene analysis utilizing pre-treatment data between responders and non-responders (**Figure 2A**; **Supplementary Table 4**). Significant differential expression was not observed in any genes, including *TNFSF13B* and *TNFRSF13C* (FDR>0.05, **Supplementary Figure 3**). However, when we tested the potential difference at the pathway level by gene set enrichment analysis (GSEA) using pre-treatment expression data, there was the most enrichment in the pathways related to B cell proliferation (**Figure 2B**). One of the most significant pathways, the “B cell proliferation” pathway, contained 100 genes, of which 7 had *p*-values below 0.05, all showing higher expression in non-responders (**Figure 2C**). The average expression of these 100 genes was significantly lower in both responders and non-responders compared to healthy controls (*p* < 0.05); however between responders and non-responders, responders showed higher expression with borderline significance (*p*=0.049, **Figure 2D**). This gene module expression was still higher in the non-responders than in the responders after six months (*p*=0.03). The seven genes shown in **Figure 2C** also showed similar dynamics during treatment (**Supplementary Figure 4**). We also found that the average expression of these seven genes could predict the treatment response with fair accuracy (AUC = 0.76, **Figure 2E**). We tested the association between the “B cell proliferation” pathway and various phenotypes and found negative correlation with PSL dose, white blood cell count, C4 and positive correlation with type I IFN pathway (**Supplementary Table 5**).

### 3.3 Memory B cells were abundant in non-responders before treatment

**Figure 3:**
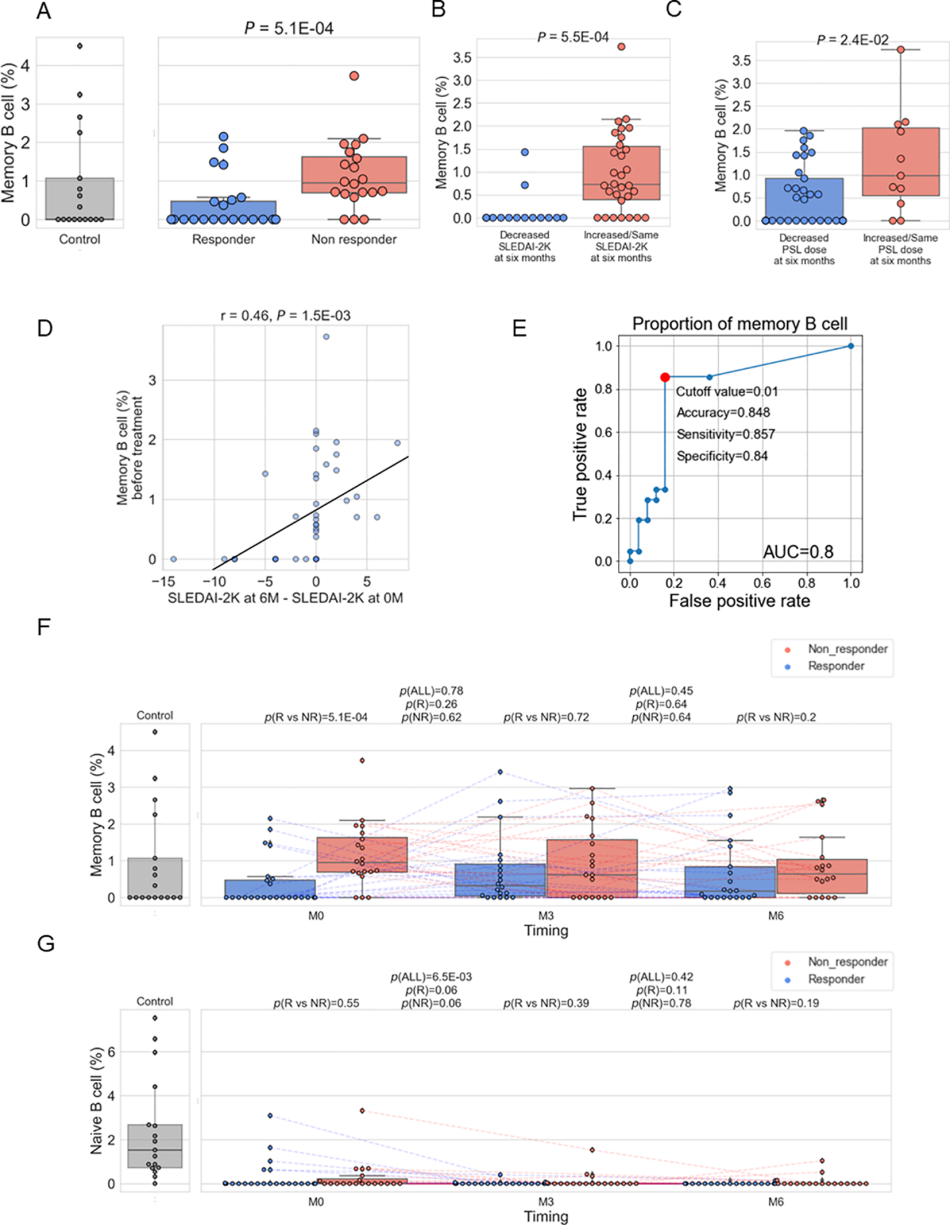
**(A–C)**. Box plots comparing the proportion of memory B cells to total white blood cells between responders and non-responders as defined in Methods **(A)**, between patients with a decreased SLEDAI-2K [(SLEDAI-2K at six months) – (SLEDAI-2K before treatment) < 0] and those with an increased SLEDAI-2K **(B)**, and between patients with a decreased prednisolone (PSL) dose ((PSL dose at six months) / (PSL dose before treatment) < 1) and those with an increased PSL dose **(C)**. **(D)** Relation between memory B cell (%) before treatment and [(SLEDAI-2K at six months) – (SLEDAI-2K before treatment)] **(E)** ROC curve for non-response to belimumab. Red plots indicate the cut-off point at the highest accuracy for predicting non-responders. **(F, G)** Box plots comparing the proportion of memory B cells **(E)** and naïve B cells **(F)** between responders and non-responders and their time course. Each dot represents each specimen. Red and blue boxes represent NR and R groups. As for the P values, please refer to the legend of **Figure 1**.

To investigate the immune cell subtypes associated with resistance to belimumab, we employed immune cell type enrichment analysis to estimate the proportions of each immune cell subset in each specimen. We have confirmed these estimates showed high correlations with clinical measurements (**Supplementary Figure 5**). Correlation analysis between B cell subtypes (memory B cell, naïve B cell, plasma cell) and the mean expression of the 100 genes in the B cell proliferation pathway utilizing SLE patient-derived specimens (n=123) showed the highest correlation with memory B cell (**Supplementary Table 6**). Therefore, we first compared the proportion of memory B cell types of pre-treatment data between responders and non-responders, finding a higher proportion in non-responders (*p*=5.1×10^-4^) (**Figure 3A**). This association was also confirmed when a responder was defined as having a SLEDAI-2K score of 4 or less at six months, instead of the definition of less than 4 described in the Methods (**Supplementary Figure 6A**). We further divided the patient groups based on the decrease of SLEDAI-2K and PSL reduction at six months post-treatment, finding higher memory B cell proportions in patients with [(SLEDAI-2K at six months – SLEDAI-2K at baseline)] ≥ 0 and those unable to reduce the dosage of PSL (**Figures 3B, C**). Furthermore, the proportion of memory B cells before treatment exhibited a positive linear correlation with SLEDAI-2K increase (**Figure 3D**), and a positive linear correlation with [(PSL dose at 6 month) – (PSL dose at baseline)] (**Supplementary Figure 6B**), and [(PSL dose at 6 months)/(PSL dose at baseline)] (**Supplementary Figure 6C**), with suggestive significance. Overall, a higher proportion of memory B cells was identified as a treatment resistance factor, showing consistency across multiple response criteria.

To compare the results of memory B cells with other immune cell subtypes, we tested the difference between non-responders and responders for the remaining cell subtypes (**Table 1**). Although naïve CD4^+^ T cells showed significance (*p* =0.04) with higher expression in non-responders, this significance was lower than that observed for memory B cells. Therefore, it was suggested that an increase in memory B cells is a more prominent hallmark of treatment resistance in SLE patients. We found the proportion of memory B cells before treatment predicted the response with good accuracy (AUC=0.8) (**Figure 3E**). In order to search for alternative markers of memory B cells, correlations of the ratio of memory B cells with other traits, such as type I IFN score and clinical traits, were calculated using samples from enrolled patients. As a result, a positive correlation with type I IFN score and negative correlations with the number of white blood cells and complement C4 were observed (**Supplementary Table 7**). Among those indices, the number of white blood cells exhibited poor predictive accuracy; however, type I IFN score and C4 had fair predictive accuracy (**Supplementary Figure 7**).

### 3.4 Belimumab had no effect on the abundance of memory B cells in contrast to naïve B cells

Next, changes in the B cell subtype due to treatment with belimumab were examined. It was found that there were no significant changes in the proportion of memory B cells due to belimumab administration (**Figure 3F**). Non-responders exhibited the tendency of a higher proportion of memory B cells compared to responders across the treatment time course. Additionally, the impact of belimumab administration on naïve B cells and plasma cells was investigated. A significant decrease was observed in naïve B cells at three months of treatment, with no change observed from 3 to 6 months of treatment (**Figure 3G**). On the other hand, no significant changes were observed in plasma cells (**Supplementary Figure 8**). These findings indicate potential bias in the effects of belimumab treatment across B cell subtypes.

### 3.5 Upregulated genes in memory B cells of non-responders

**Figure 4:**
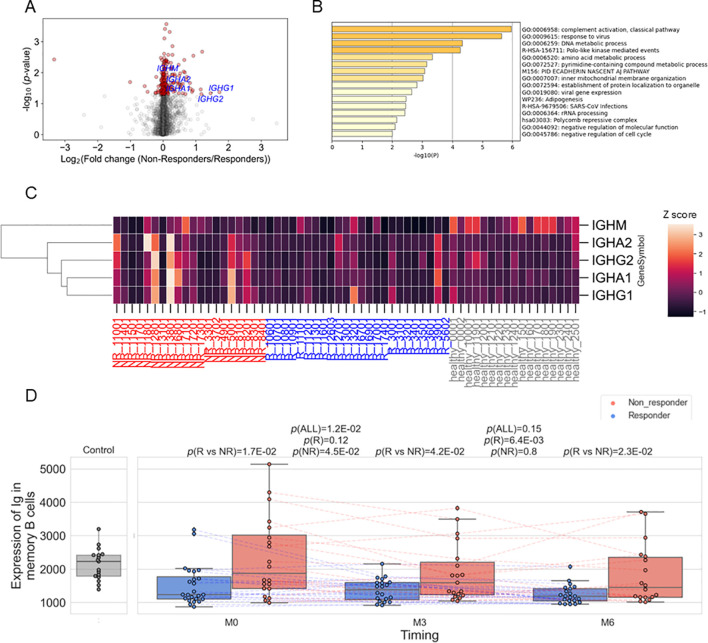
**(A)** Volcano plots showing differentially expressed genes between responders (R) and non-responders (NR) before treatment in memory B cells. Red dots represent *p*<0.05. **(B)** The results of enrichment analysis of genes upregulated in non-responders before treatment in memory B cells. **(C)** Heatmap showing the expression of the five immunoglobulin genes (*IGHM, IGHA2, IGHG2, IGHA1*, and *IGHG1*) in memory B cells in each sample. Expression was standardized across all samples, and the Z scores were shown. **(D)** Box plots comparing the average expression of the five immunoglobulin genes and their time course. Each dot represents each specimen. Red and blue boxes represent NR and R groups. As for the P values, please refer to the legend of **Figure 1**.

To characterize the transcriptomic feature in memory B cells of non-responders, we estimated each gene’s expression levels in each specimen’s memory B cells using the immune cell type enrichment analysis and compared between non-responders and responders. Although none of the genes showed significance with FDR < 0.05, 225 genes showed nominal differences (*p* < 0.05), with 164 of them being higher in non-responders (**Figure 4A**; **Supplementary Table 8**). Enrichment analysis of these 164 genes revealed the strongest enrichment in the complement activation pathway (*q*-value = 0.02) (**Figure 4B**; **Supplementary Table 9**). It was found that immunoglobulin genes (*IGHM, IGHA2, IGHG2, IGHA1*, and *IGHG1*) contributed to the enrichment (**Supplementary Table 9**, **Figure 4C**), and all the five genes were upregulated in non-responders than in responders (**Figure 4A**, **Supplementary Table 8**). Hereafter, we call these five genes as Ig. We calculated the average expression levels of Ig in memory B cells for each sample and found that non-responders had higher levels of Ig compared to responders (**Figure 4D**). Although the expression levels decreased following belimumab administration, non-responders showed higher levels than responders throughout the treatment course, up to six months post-administration.

**Figure 5:**
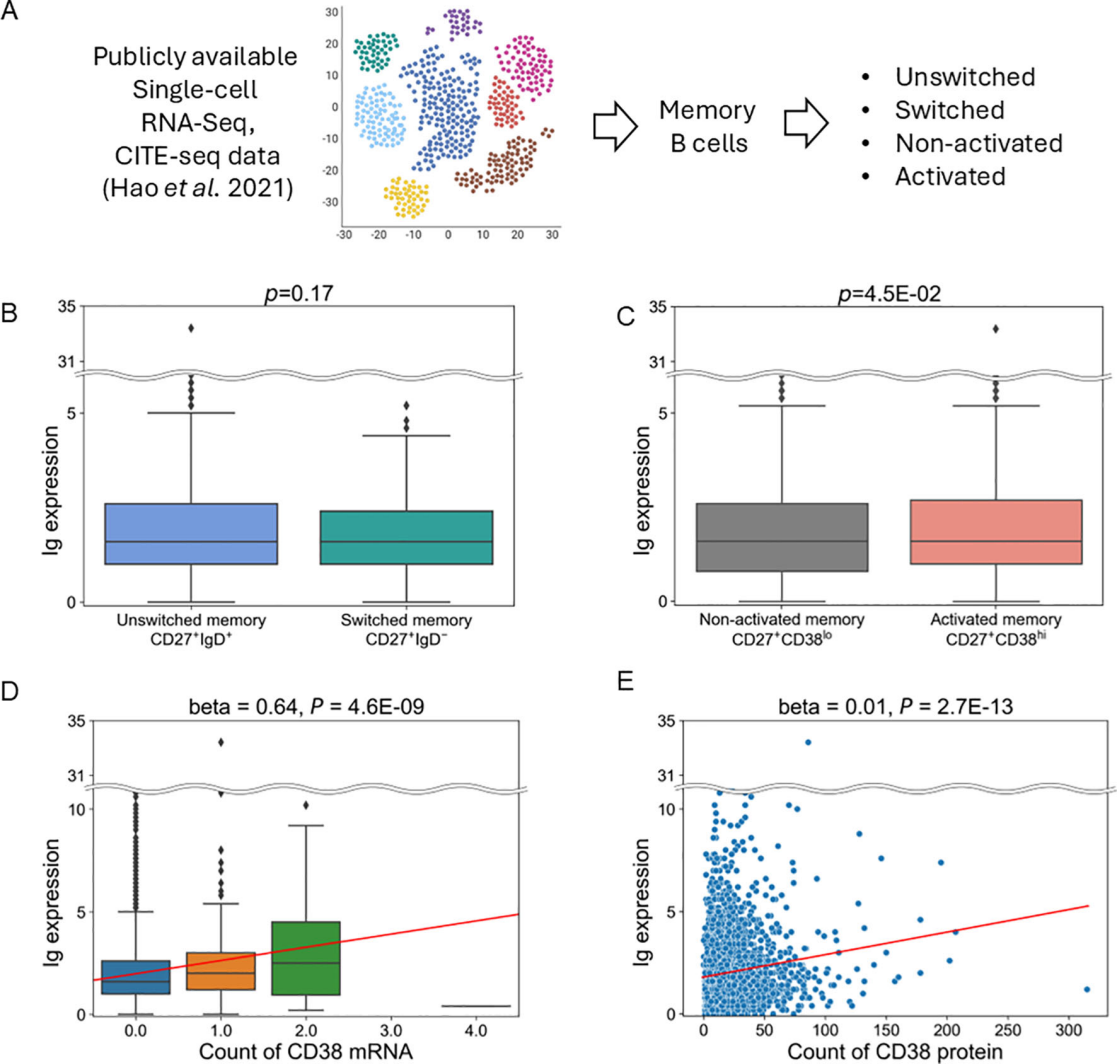
Analysis of publicly available single-cell RNA and CITE-Seq data. **(A)** Schematic of this analysis. **(B, C)** Comparison of Ig expression between switched and unswitched memory B cells **(B)** or between activated and non-activated memory B cells **(C)**. **(D, E)**. Association between the expression of Ig and CD38 mRNA levels **(D)** and CD38 protein levels **(E)** in memory B cells. The horizontal line represents the unique molecular identifiers (UMI) count of CD38 mRNA **(D)** and protein **(E)** derived from scRNA-seq and CITE-Seq data. Red lines indicate the line regressed by linear regression analysis.

Finally, we aimed to identify cell populations that express Ig in memory B cells and their related cell types. We first attempted to perform analysis utilizing single-cell data derived from patients with SLE (34), but there weren’t a sufficient number of cells to perform analysis (data not shown). Alternatively, we used publicly available single-cell data of human PBMCs (**Figure 5A**) (29). When comparing populations between unswitched memory B cells (CD27^+^IgD^+^) and switched memory B cells (CD27^+^IgD^-^), there was no difference in Ig expression (**Figure 5B**). However, Ig expression was higher in activated memory B cells (CD27^+^CD38^hi^) than in non-activated memory B cells (CD27^+^CD38^lo^) (**Figure 5C**). CD38 is a surface glycoprotein that serves as an activation marker on B cells (35). Furthermore, Ig expression levels showed a positive correlation with the expression of CD38 mRNA as well as CD38 protein (**Figures 5D, E**). Therefore, these results indicate that activated memory B cells are the primary source of Ig expression.

## 4 Discussion

We identified transcriptomic and cellular characteristics related to the response of belimumab. The B cell proliferation pathway was upregulated in non-responders, which was associated with an increase in memory B cells. The proportion of those cells was found to be higher in non-responders before treatment. Belimumab did not induce significant changes in this transcriptomic and cellular feature during the treatment time course. More precisely, only 4 genes decreased their expressions after three months of treatment (**Figure 1J**) and no differentially expressed genes were detected between three months after and six months after treatment (**Supplementary Table 3**). Consequently, the difference in memory B cell proportion between responders and non-responders persisted after six months of treatment. In contrast, the number of naïve B cells decreased rapidly with belimumab administration in all patients (responders + non-responders, **Figure 3G**), consistent with previous reports (12, 36). These results suggest a potential bias in the effect of belimumab among B cell subtypes.

What drives memory B cell proliferation? We found memory B cells, B cell proliferative signals and type I IFN were all found to be positively correlated with each other (**Supplementary Tables 5**–**7**). This suggests that an increase in type I IFN may contribute to the activation of proliferative signaling and subsequent increase in memory B cells. Indeed, a recent report has shown that type I IFNs are associated with memory B cell proliferation in chronic infectious conditions (37).

We found the expression of Ig was higher in memory B cells in non-responders than in responders, which persisted after six months. Our data-driven approach defined “Ig” as a gene set that contains not only IGHG/A, markers for class switching, but also IGHM, a marker for non-class switching. This explains why the expression of such defined Ig did not differ between unswitched and switched memory B cells (**Figure 5B**). Our single-cell analysis using publicly available data indicated Ig is predominantly expressed by activated memory B cells, defined as CD27^+^CD38^hi^ (**Figures 5C–E**). These cells can also be referred to as plasmablasts. Together, our analysis suggests that belimumab may have limited effects on these activated memory B cells. Further studies are warranted to confirm the involvement of activated memory B cells and their Ig expression during belimumab treatment.

Based on the results of this study, it is crucial to establish criteria for predicting responsiveness to belimumab in clinical settings. We have discovered that a higher proportion of memory B cells predicts non-responsiveness with high accuracy (**Figure 3E**). However, promptly assessing the proportion of memory B cells in clinical settings is challenging. We explored the substitute markers and found a significant correlation between higher levels of memory B cells and lower levels of C4 and white blood cells, and lower levels of C4 predict the treatment response with fair accuracy (**Supplementary Figure 7**).

Then, what alternative treatments could be considered for patients who were predicted to be poor responders to belimumab? Rituximab may help decrease the number of memory B cells. Additionally, since the number of memory B cells showed a positive correlation with the type I IFN score, anifrolumab (anti-type I IFN receptor monoclonal antibody) could be one of the candidates. Furthermore, although not currently approved for SLE, Janus kinase (JAK) inhibitors are also potential candidates since JAK is one of the essential components of IFN signaling (38, 39). They would modulate B cell activation, differentiation, and functions by reducing STAT3 signaling, leading to decreased switched memory B cell formation and decreased immunoglobulin production, as shown in the previous *in-vitro* and clinical study (40). Lastly, continuous use of belimumab, irrespective of initial treatment response, may aid in reducing memory B cells, as reported in a study demonstrating that seven-year treatment depleted the number of memory B cells (41). Further clinical research is warranted to establish a treatment strategy for SLE with higher number of memory B cells.

There are several conflicting results compared to previous reports. Firstly, it was found that patients with higher disease activity before treatment showed poor responsiveness to belimumab (**Table 1**), which contradicts previous findings (8). Differences in the disease activity of the analyzed patients could partly explain this. While the previous report focused on patients with high disease activity, our study targeted patients with low to moderate activity. Another potential explanation is the difference in treatment response definition. While the previous study employed the SLE Responder index, which includes the changes in SLEDAI, the present study employed the criterion of responders when SLEDAI-2K < 4 after six months. We will discuss this response criteria issue further in the next paragraph. Secondly, patients with high SDI showed good responses, contrary to previous findings (9). This could be explained by the confounding effect of disease duration; responders tended to show more extended disease duration periods (**Table 1**). Third, in contrast to several reports indicating a transient increase in memory B cells after Belimumab treatment initiation (42, 43), there was no significant change in the proportion of memory B cells (**Figure 3F**). However, this phenomenon could not be replicated in another study (12), and there are suggestive increases, especially in responders, after 3 months of treatment initiation.

Based on the primary definition of response criteria in our study (SLEDAI-2K <4 at six months), responders showed low disease activity before treatment with minimal changes in clinical indices during the six months (**Figures 1E–I** and **Table 1**). Additionally, some non-responders showed improvements in certain parameters, such as increased C3 levels and decreased anti-dsDNA antibody titers (**Figures 1F, H** and **Table 1**). These observations reflect an inherent challenge in SLE clinical research: defining treatment response in patients with low to moderate disease activity is particularly challenging due to the limited margin for improvement in conventional metrics. Despite these limitations in response definition, our key finding regarding memory B cell proportions remains consistent. The association between higher memory B cell proportions and non-response was confirmed across multiple response criteria, including definitions based on SLEDAI-2K changes (**Figure 3B**) or PSL dosage reductions (**Figure 3C**). Furthermore, we demonstrated a linear correlation between memory B cell proportions and SLEDAI-2K changes (**Figure 3D**), supporting the biological relevance of this association regardless of response definition. In **Figure 2D**, the average expression levels of the 100 genes in the B cell proliferation pathway were higher in healthy controls compared to both non-responders and responders (SLE patients). This could be attributed to the effects of treatments, such as steroids or immunosuppressants. Although non-responders showed higher expression levels in the B cell proliferation pathway compared to responders, their levels remained lower than those of healthy controls.

There are several limitations to this study. Firstly, the proportion of immune cell subset and gene expression in each cell subset were not experimentally determined, such as through flow cytometry or RNA-Seq in each cell subtype but were based on estimation by cell type enrichment analysis. Nevertheless, this estimation showed a good correlation with clinical measurements (**Supplementary Figure 5**). Secondly, our cohort followed patients for six months, but longer-term observation may yield additional perspectives, as discussed above (41). Thirdly, none of the Ig components showed significance at the conservative threshold (FDR <0.05). Therefore, the contribution of each gene contribution should be interpreted with caution. However, the enrichment analysis, which evaluates the genes as a ranked collection rather than individual components, still yields statistically significant results (*q*-value =0.02, **Figure 4B**; **Supplementary Table S9**). Fourth, our analysis using single-cell RNA-seq and CITE-seq data was derived from non-SLE subjects, which warrants further study to identify the cellular source of Ig expression. Another limitation is the relatively small sample size compared with previous clinical studies. Considering the heterogeneity of SLE, confirmation of the obtained results in more extensive and independent cohorts is warranted.

## Data Availability

The gene expression dataset supporting the conclusions of this article is available in the Zenodo repository, https://doi.org/10.5281/zenodo.14557188. These data can be downloaded without restriction.
