# Supplementary material for: Memory B cells and their transcriptomic profiles associated with belimumab resistance in systemic lupus erythematosus in the maintenance phase
Source: Front Immunol. 2025 Feb 5;16:1506298. doi: 10.3389/fimmu.2025.1506298 (PMC11835923; doi:10.3389/fimmu.2025.1506298)
Supplement: Supplementary file 1 [file DataSheet1.zip › Supplementary_Figures.docx]

**
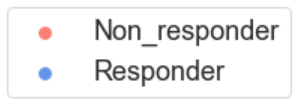
**

**
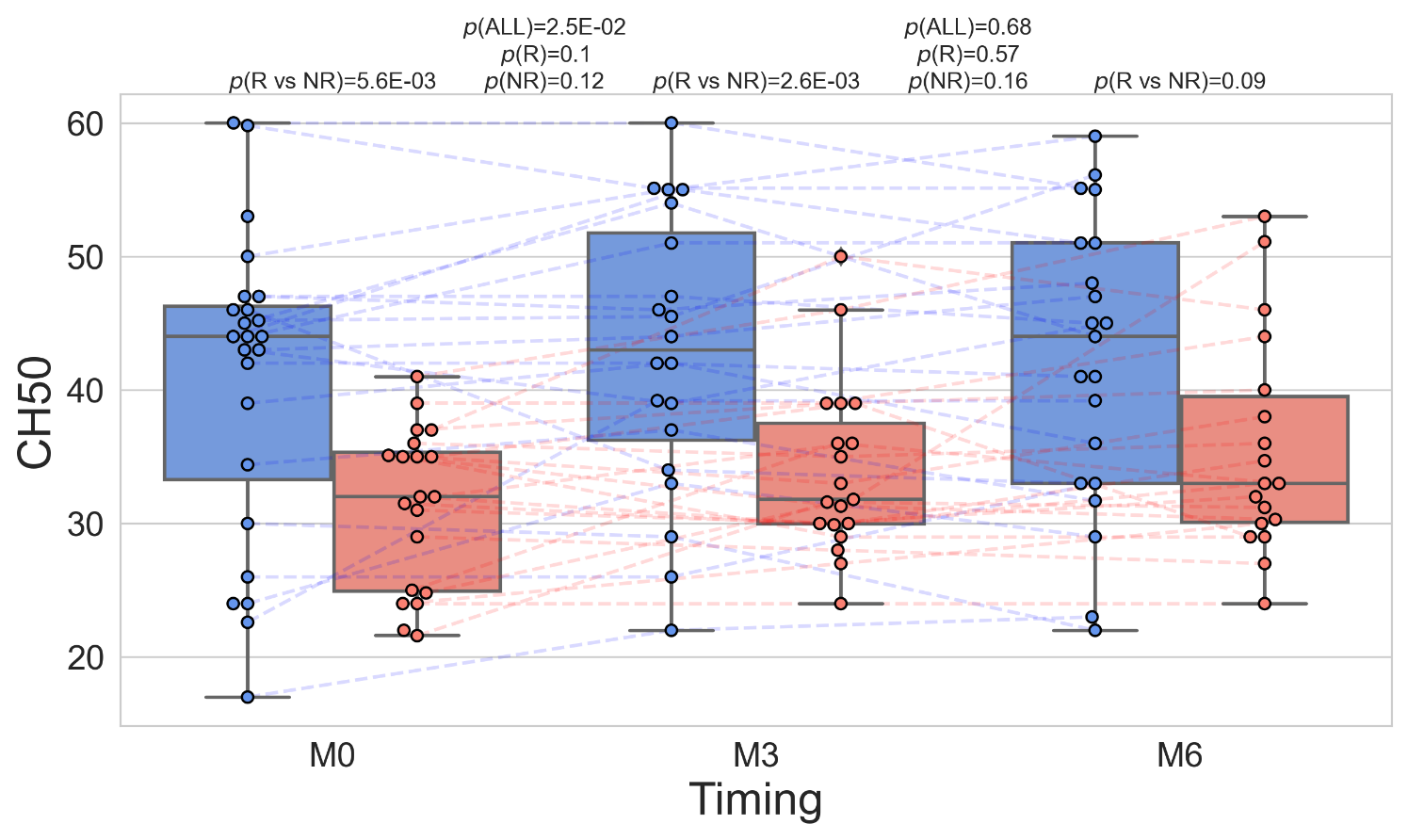
**

**Supplementary Figure 1.** Box plots comparing the level of CH50 between responders and non-responders and its time course. "M0" stands for before treatment, "M3" for 3 and "M6" for six months after treatment. As for the *P*-values, please refer to the legend of Figure 1.


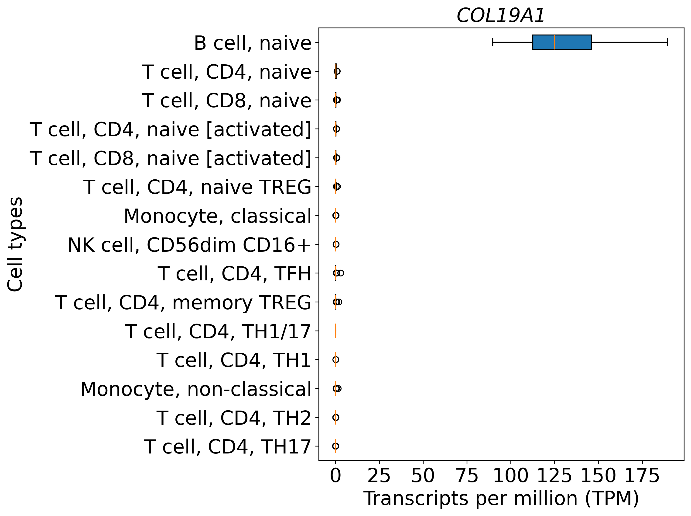
**
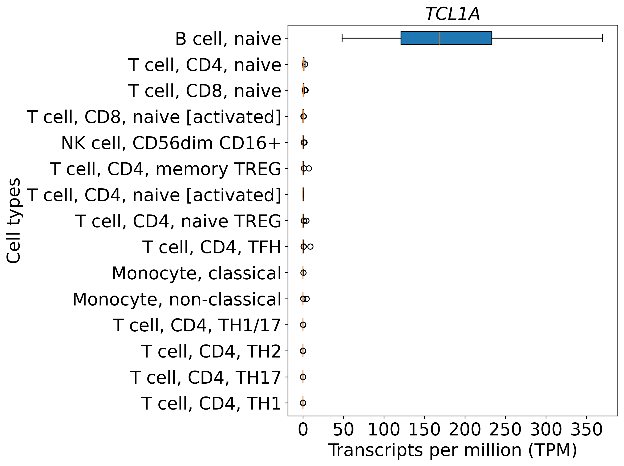
**

**B**

**A**


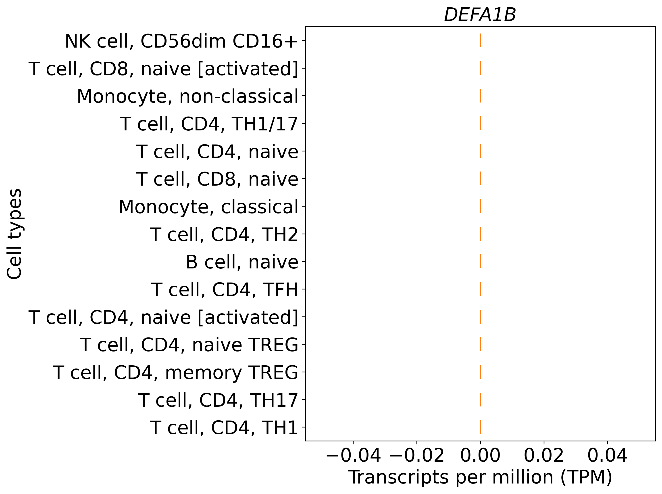
**
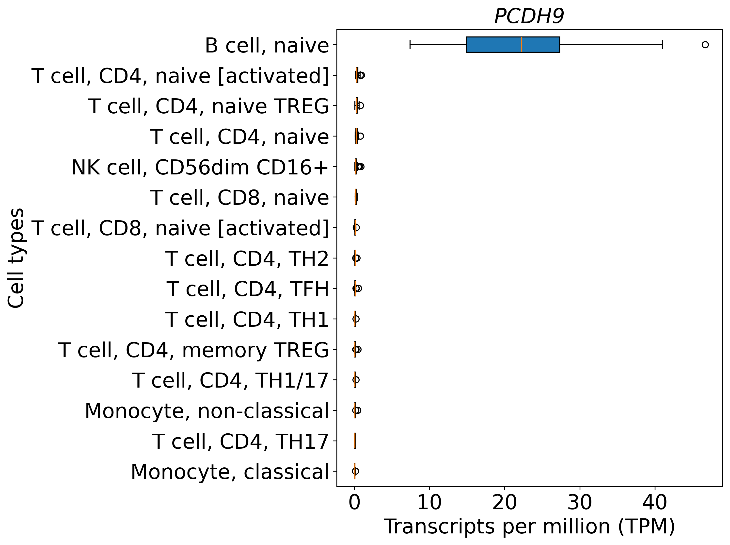
**

**D**

**C**

**Supplementary Figure 2.** Comparison of gene expression level across immune cell subtypes using the DICE database. *DEFA1B* (Panel D) was below the sensitivity limit across all cell types.


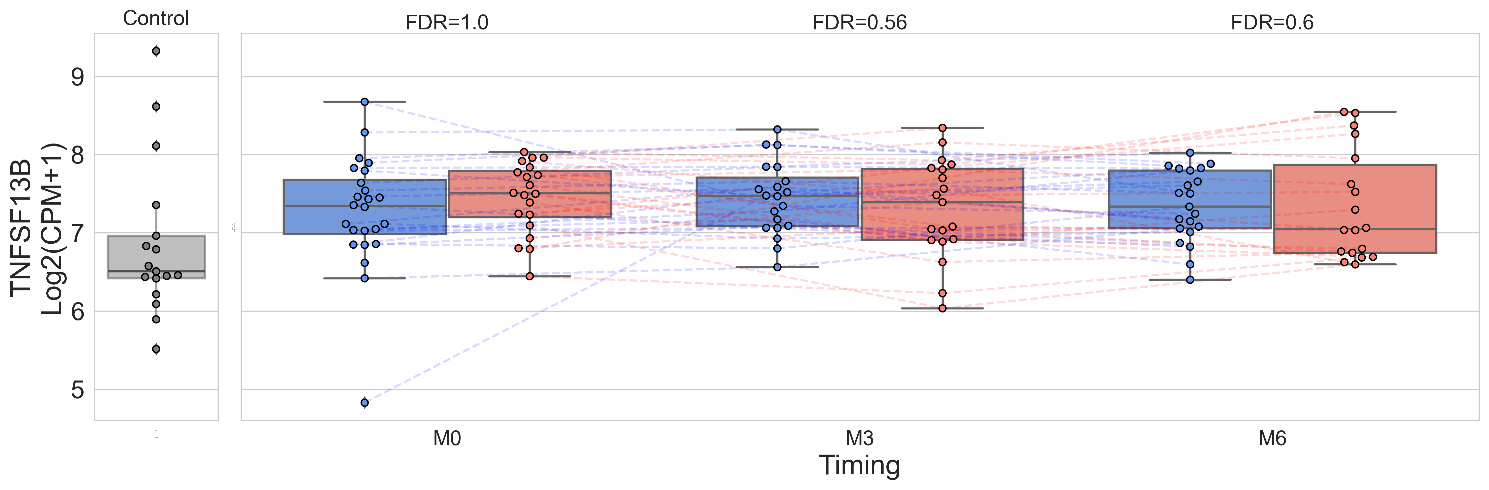


**A**

**B**

**
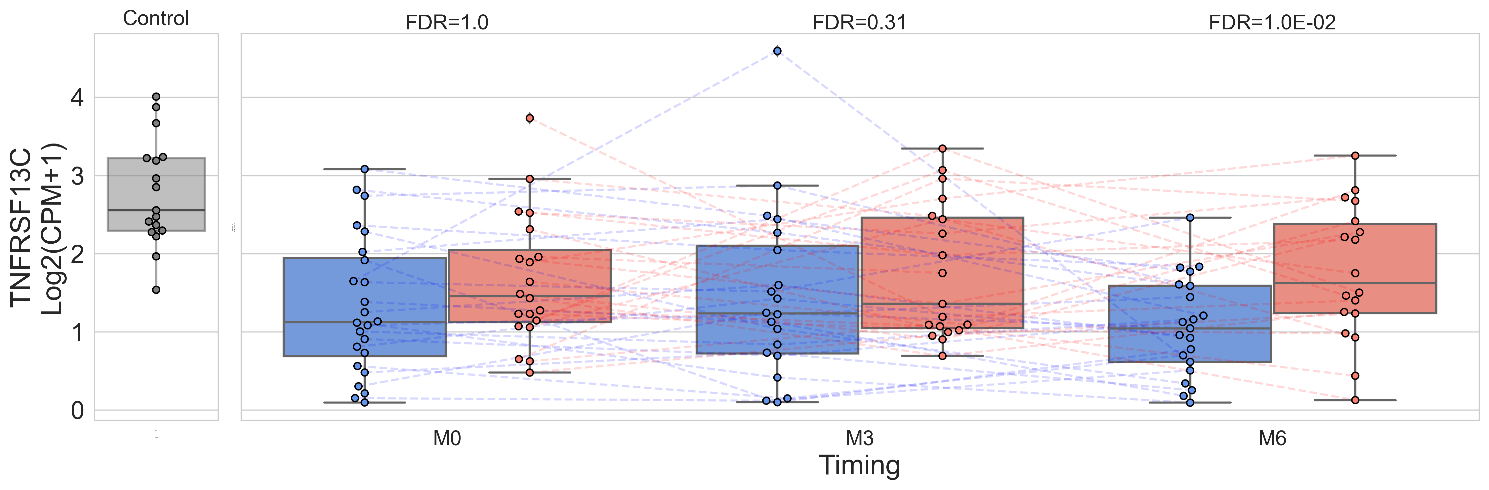
**

**Supplementary Figure 3.** Comparison of the *TNFSF13B* (**A**) and the *TNFRSF13C* (**B**) expression in healthy individuals, responders, non-responders, and their time course. CPM: count per million.

**
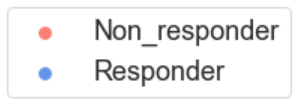
**


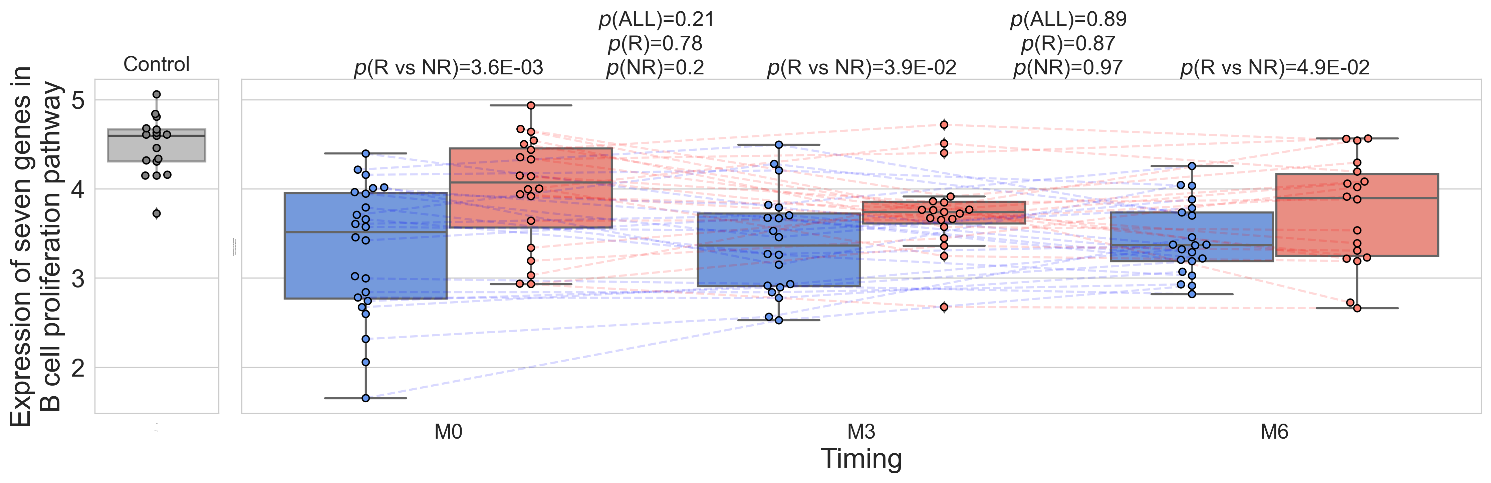


**Supplementary Figure 4.** Box plots comparing the average expression of the seven genes (Figure 2C) between responders and non-responders and their time course. As for the *P*-values, please refer to the legend of Figure 1.

**C**

**B**

**A**

**
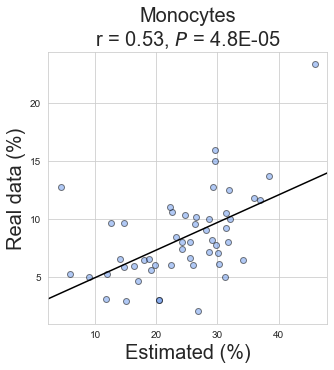

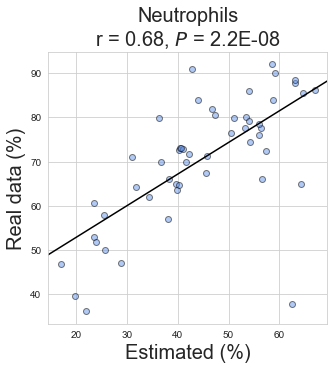

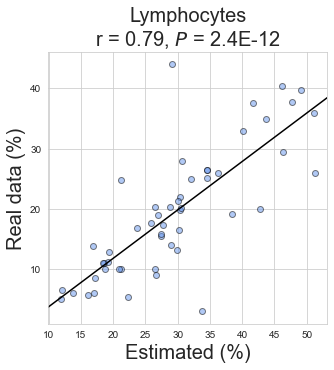
**

**Supplementary Figure 5.** Association between the clinical values and the estimated values by the immune cell type enrichment analysis. The estimated proportion of lymphocytes is the sum of 12 cell subtypes which can be classified into lymphocytes.

**C**

**B**

**A**

**
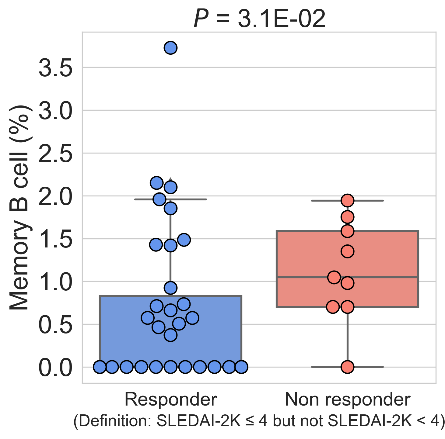

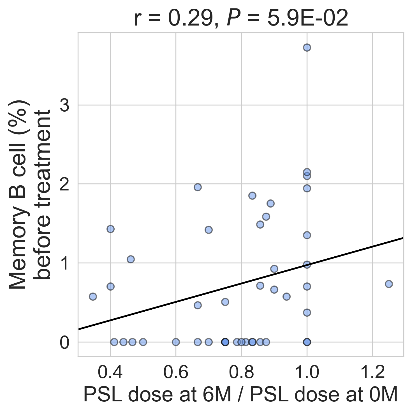
**
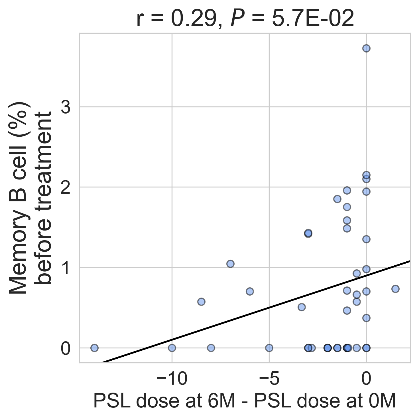


**Supplementary Figure 6.** (**A**) Box plots comparing the proportion of memory B cells to total white blood cells between responders and non-responders, defining SLEDAI-2K ≤ 4 at 6-months as responders. (**B,C**) Relation between memory B cell (%) before treatment and ((PSL dose at six months) – (PSL dose before treatment)) (**B**), and ((PSL dose at six months) / (PSL before treatment)) (**C**). PSL:prednisolone

**
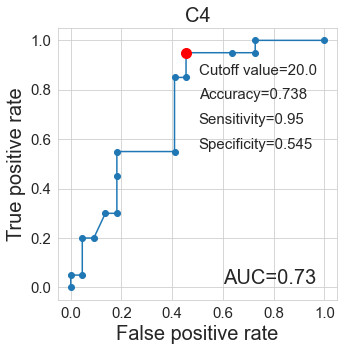

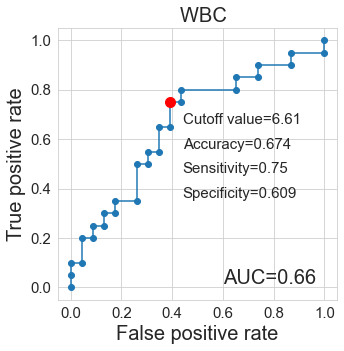

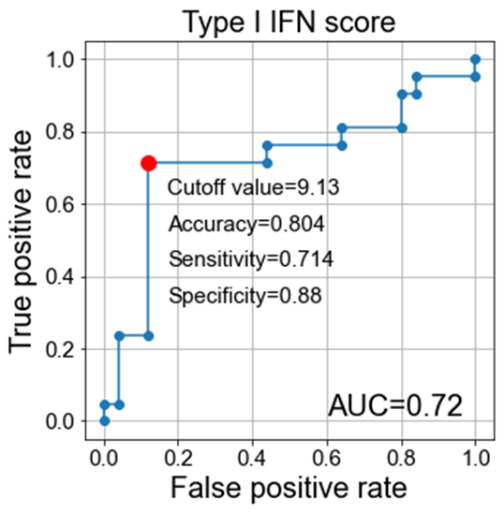
**

**B**

**A**

**C**

**Supplementary Figure 7.** ROC curves for non-responders to belimumab. Red plots indicate the cut-off point at the highest accuracy for predicting non-responders. Basically, we calculated true/false positive rate and cutoff value to distinguish non-responders from responders; however, as for white blood cell count and C4, because the values were lower in non-responders, we calculated cutoff values to detect responders from non-responders.

**
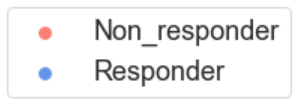
**


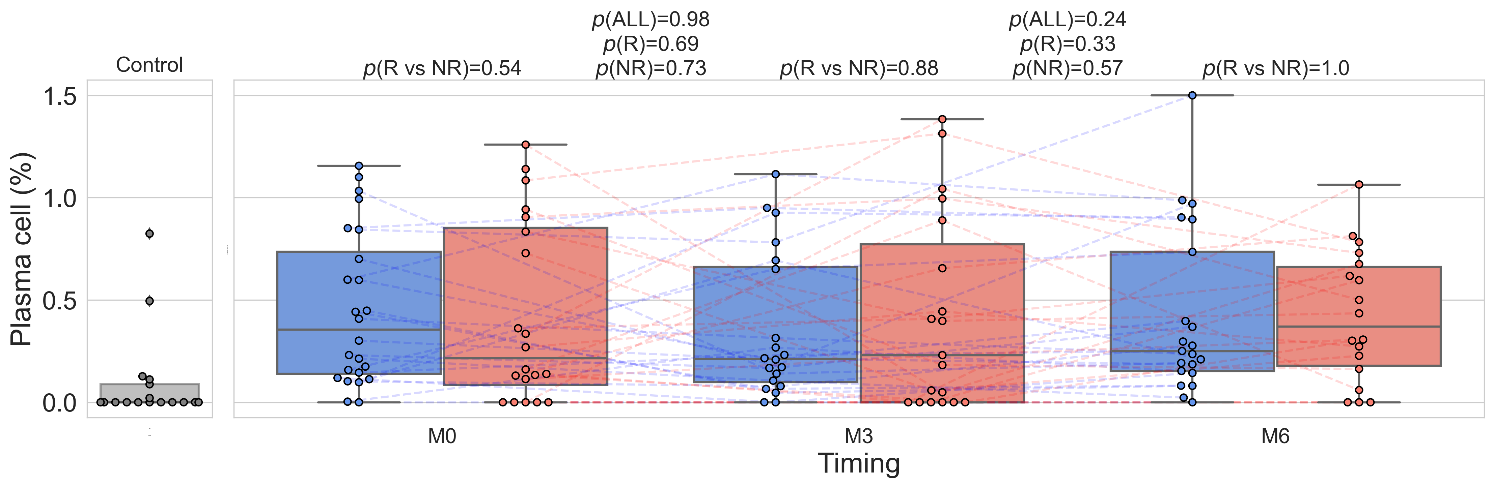


**Supplementary Figure 8.** Box plots comparing the proportion of plasma cells to total white blood cells between responders and non-responders and their time course. As for the *P*-values, please refer to the legend of Figure 1.
